# HIC-5 in cancer-associated fibroblasts contributes to esophageal squamous cell carcinoma progression

**DOI:** 10.1038/s41419-019-2114-z

**Published:** 2019-11-18

**Authors:** Xuanling Du, Qiping Xu, Duyi Pan, Dongke Xu, Baolin Niu, Wenting Hong, Rui Zhang, Xiaobo Li, Shiyao Chen

**Affiliations:** 1 Department of Gastroenterology and Hepatology, Zhongshan Hospital, Fudan University, Shanghai, 200032 P.R. China; 2 Department of Internal Medicine, John H. Stroger Jr. Hospital of Cook County, Chicago, IL 60612 USA; 3 Department of Physiology and Pathophysiology, School of Basic Medical Sciences, Fudan University, Shanghai, 200032 P.R. China; 4 Center of Evidence-Based Medicine, Fudan University, Shanghai, 200032 P.R. China

**Keywords:** Cancer microenvironment, Oesophageal cancer

## Abstract

Esophageal squamous cell carcinoma (ESCC) remains one of the most common malignancies in China and has a high metastasis rate and poor prognosis. Cancer-associated fibroblasts (CAFs), a prominent component of the tumor microenvironment, can affect tumor progression and metastasis, but the underlying mechanism remains unclear. There are no studies that explore the role of hydrogen peroxide-inducible clone 5 (HIC-5) in ESCC or compare the role of HIC-5 in CAFs and adjacent noncancerous normal fibroblasts (NFs). In this study, we isolated primary CAFs and NFs from ESCC patients. HIC-5 was highly expressed in CAFs from the tumor stroma of human ESCC patients. HIC-5 knockdown in CAFs inhibited the migration and invasion of ESCC cells in vitro. Supernatant CCL2 levels of CAFs were significantly higher after TGF-β stimulation and lower after knocking down HIC-5 expression, independent of TGF-β treatment. HIC-5 knockdown in CAFs led xenograft tumors derived from ESCC cells mixed with CAFs to present more regular morphology, express higher CDH1, and lower CCL2. Further RNA-seq data showed that HIC-5 has distinct biological functions in CAFs vs. NFs, especially in cell movement and the Rho GTPase signaling kinase pathway, which was verified by wound-healing assays and western blotting. An ESCC tissue microarray revealed that increased HIC-5 expression in the tumor stroma was associated with positive lymph node metastasis and a higher TNM stage. In summary, we identified that stromal HIC-5 was a predictive risk factor for lymph node metastasis in human ESCC and that CAF-derived HIC-5 regulated ESCC cell migration and invasion by regulating cytokines and modifying the ECM.

## Introduction

Esophageal cancer, one of the most common malignancies, has a global incidence and mortality ranking of 7th and 6th, respectively, and it has the third lowest 5-year relative survival rate of all cancers.^1^ China, one of the countries with the highest esophageal cancer incidence and mortality rates, has reported 375,000 cases of death annually.^2^ Esophageal adenocarcinoma (EAC) and esophageal squamous cell carcinoma (ESCC) are two major esophageal cancer subtypes. ESCC accounts for 88.84% of all cases in China, which is different from the pattern in western countries where EAC is much more common.^3,4^ The disease is metastatic in over 60% of newly diagnosed cases, which is one of the principal reasons why the overall 5-year survival rate of esophageal cancer is <15%.^5^ Despite early detection efforts, lymph node metastasis/recurrence rates range from 0% to 62%, even in T1 ESCC patients.^6^ Studying the molecular mechanism and identifying biomarkers that predict ESCC metastasis will provide insight for developing targeted therapies that will hopefully lead to a better prognosis.

Tumor microenvironment (TME) is an important contributor to cancer progression and metastasis.^7^ TME, also known as tumor stroma, consists of the immune cells, capillaries, basement membrane, activated fibroblasts, and extracellular matrix (ECM) surrounding cancer cells.^8^ With the coevolution of tumor cells and stroma cells, the TME can be transformed into a pathogenic state, accelerating the invasion of tumor cells, and creating the environment for distant migration.^9,10^ As a prominent component of the TME, cancer-associated fibroblasts (CAFs), also known as tumor-associated fibroblasts (TAFs), play a complex role. Although both tumor-promoting and tumor-inhibiting characteristics have been observed in the literature, CAFs are generally considered to contribute to cancer progression and metastasis.^8,11–13^ CAFs release cytokines and growth factors into the ECM as well as circulate to enhance cancer metastasis, even at distant sites.^14^ ECM remodeling and CAF metabolic reprogramming also contribute to the symbiotic relationship with tumor cells.^15,16^

Hydrogen peroxide-inducible clone 5 (HIC-5), also known as transforming growth factor beta-1-induced transcript 1 (TGFβ1i1), was first identified to be induced by H_2_O_2_ and TGF-β1.^17^ With four LIM motifs, it is a closely related family member of PAXILLIN and is a focal adhesion protein that shuttles between focal adhesions and the nucleus under oxidant stimulation.^18^ HIC-5 is highly expressed in vascular and visceral smooth muscle cells in human tissue and helps regulate the contractile capability of cells and muscle differentiation.^19,20^ HIC-5 expressed in tumor cells enhances cancer invasion and migration, as shown in previous studies of breast cancer, prostate cancer, hepatocellular carcinoma, and melanoma.^21–24^ Recent studies have gradually drawn attention to the functions of HIC-5 in fibroblasts and its crosstalk with cancer cells. HIC-5 expressed in CAFs contributes to the invasion and migration of breast cancer and prostate cancer through ECM remodeling and cytokine secretion, and it is responsible for colorectal cancer tumorigenesis.^25–28^ Despite the studies above, the mechanism of how CAF-derived HIC-5 modulates tumor progression remains unclear. In addition, no study has explored the role of HIC-5 in ESCC or compared the role of HIC-5 in CAFs and normal esophageal fibroblasts.

Herein, we first examined HIC-5 expression in human ESCC. Then, we separated CAFs from human ESCC tissue and explored the role of HIC-5 in CAFs and its effect on ESCC cancer cells. We also compared the role of HIC-5 in CAFs and normal esophageal fibroblasts and investigated the possible mechanisms by which HIC-5 contributes to ESCC progression.

## Materials and methods

### Isolation of fibroblasts from human tissue

CAFs and paired normal fibroblasts (NFs) were isolated from tumor tissue and adjacent non-tumor tissue (more than 5 cm away from the tumor margin) from ESCC patients who underwent surgical resection at the Department of Thoracic Surgery, Zhongshan Hospital, Fudan University. Informed consent was obtained from all patients, and the study was approved by the Ethics Committee of Zhongshan Hospital, Fudan University. After washing with phosphate buffered saline (PBS), the tissues were cut and minced into small pieces (1–2 mm^3^) and seeded in a culture flask with Dulbecco’s modified Eagle’s medium (DMEM; Thermo Fisher Scientific, Waltham, MA, USA) containing 10% fetal bovine serum (FBS). Fibroblasts grew outwards from the explants and reached 80% confluence after 2 weeks. Homogeneous CAFs and NFs were cultured in fibroblast medium (Sciencell, Carlsbad, CA, USA) for later experiments.

### Cell culture

The human ESCC cell lines KYSE150 and TE1 were purchased from the Cell Bank of the Chinese Academy of Sciences (Shanghai, China), where STR profile authentication and mycoplasma contamination test were also performed. Tumor cells were cultured in DMEM with 10% FBS, 100 U/mL penicillin, and 100 μg/mL streptomycin at 37 °C in a humidified cell incubator with 5% CO_2_. For TGF-β stimulation, fibroblasts were cultured to 70–80% confluence, starved for 24 h in serum-free culture medium, and then treated with 10 ng/mL TGF-β1 (Selleck Chemicals, Houston, TX, USA) for the indicated times.

### Immunohistochemistry staining

Immunohistochemistry staining was performed as described previously.^29^ Sections were incubated with a rabbit anti-HIC-5 antibody (Proteintech, Rosemont, IL, USA) and anti-αSMA (Sigma-Aldrich, St. Louis, MO, USA) at 4 °C overnight. Then, the slides were incubated with a horseradish peroxidase (HRP)-conjugated anti-rabbit antibody at room temperature for 90 min, visualized with DAB, and counterstained with hematoxylin.

### Immunofluorescence staining

Immunohistochemistry staining was performed as described previously.^29^ The slides were incubated with primary antibodies, namely, anti-αSMA (Sigma-Aldrich, St. Louis, MO, USA), anti-vimentin (Cell Signaling Technology, Danvers, MA, USA), anti-FN1 and anti-HIC-5 (Proteintech, Rosemont, IL, USA), at 4 °C overnight, followed by incubation with a fluorescent secondary antibody at room temperature for 90 min. The nuclei were visualized using DAPI, and F-actin was visualized using phalloidin (Cell Signaling Technology, Danvers, MA, USA). Representative images were acquired with a fluorescence microscope.

### Quantitative real-time reverse transcriptase polymerase chain reaction (qRT-PCR)

The total RNA was isolated from cells using TRIzol reagent (Invitrogen, Carlsbad, CA, USA) according to the manufacturer’s instructions. cDNA was synthesized using Prime Script RT reagent kit (Takara Bio Inc., Otsu, Shiga, Japan). qRT-PCR was performed to quantify expression of genes using SYBR Green PCR kit (Yeasen, Shanghai, China), with 36B4 used as an endogenous reference. Primer sequences used are listed in the Supplementary Table S1.

### Western blotting

Total protein was isolated from cells using RIPA Lysis Buffer (Beyotime, Shanghai, China) containing phenylmethanesulfonyl fluoride (PMSF) and phosphatase inhibitor cocktail (PIC). After quantifying the protein concentration using a BCA Protein Assay Kit (Beyotime, Shanghai, China), western blot analysis was performed as described previously.^29^ GAPDH protein was used as a loading control to normalize protein loading. The primary antibodies used were against HIC-5, GAPDH, FN1 (Proteintech, Rosemont, IL, USA), αSMA (Abcam, Cambridge, MA, USA), p-SMAD2 (Ser465/467), p-SMAD3 (Ser423/425), SMAD2, SMAD3, and RAC1 (Cell Signaling Technology, Danvers, MA, USA).

### RNA interference

Small interfering RNA (siRNA) targeting HIC-5 (siHIC-5) and scrambled siRNA (siNC) as a control were transfected into CAFs and NFs for 6 h using Lipofectamine 3000 Reagent (Thermo Fisher Scientific, Waltham, MA, USA) according to the manufacturer’s instructions. The cells were collected for further experiments after 48 h of continuous culture. The HIC-5 siRNA sequence was as follows: 5′-GCAGCAGCTTCTTCGAGAA-3′ (Ribobio, Guangzhou, Guangdong, China).

### Transwell migration and invasion assay

For cell migration assays, 1 × 10^6^/mL tumor cells were cultured in serum-free DMEM and treated with 10 μg/mL mitomycin C (Selleck Chemicals, Houston, TX, USA) for 2 h. Then, the cells were seeded into Transwell chambers (8-μm pore size; Corning, Corning, NY, USA). Next, 3 × 10^4^ CAFs were seeded into the lower chambers. After co-culture for 12 h, cells on the opposite side of the chambers were fixed with 4% paraformaldehyde and stained with 0.5% crystal violet for 10 min. Photographs of the membranes were taken, and the numbers of cells were counted. For cell invasion assays, the Transwell chambers were coated with diluted Matrigel (BD Biosciences, Franklin Lakes, NJ, USA). The numbers of cells were counted after co-culture for 48 h.

### Direct three-dimensional co-culture assay

In total, 8 × 10^4^ CAFs and 8 × 10^4^ tumor cells were gently mixed with 150 μL of DMEM and then with 150 μL of Matrigel at 4 °C. The mixture was added to 14-mm plates and incubated at 37 °C for 15 min to allow gel formation. The cell–gel mixtures were overlaid with 500 μL of DMEM with 10% FBS for 5 days. After co-incubation, fibroblasts were anchored to the plate surface, while tumor cells grew three-dimensionally.

### Wound-healing assay

Fibroblasts were cultured in six-well plates to confluence, and wounds were created with 10-μL pipette tips. Next, the cells were incubated in serum-free culture medium, and images of the width of the wounds were taken from five randomly selected fields at 0 h and 48 h.

### EdU assay

Cells were seeded in 96-well plates and cultured for 24 h. EdU from a Cell-Light EdU Apollo567 In Vitro Kit (Ribobio, Guangzhou, Guangdong, China) was used for labeling. The cells were fixed with 4% paraformaldehyde and then subjected to EdU detection per the manufacturer’s instructions.

### Cell counting kit-8 assay

Tumor cells (1500/well) were seeded in 96-well plates in triplicate. Ten microliters of CCK-8 solution (Yeasen, Shanghai, China) was added to each well and incubated at 37 °C for 2 h. Then, the absorbance of the dye solution at 450 nm was measured. The optical densities of the cells were assessed 0, 24, 48, 72, and 96 h after incubation.

### Enzyme-linked immunosorbent assay (ELISA)

The same numbers of cells were cultured in serum-free culture medium for 24 h. After treatment with TGF-β1 for 24 h, the supernatants were isolated by centrifugation and stored at −80 °C. Supernatant levels of CCL2 were quantified using a Human CCL2/MCP-1 ELISA Kit (MultiSciences, Hangzhou, Zhejiang, China) according to the manufacturer’s instructions.

### RNA sequencing (RNA-seq) and data analysis

The total RNA was extracted using TRIzol reagent (Invitrogen, Carlsbad, CA, USA). After RNA enrichment, cDNA libraries were prepared using the NEBNext Ultra RNA Library Prep Kit for Illumina (NEB, Ipswich, MA, USA). After evaluating the quality of the cDNA libraries using a 2100 Bioanalyzer (Agilent Technologies, Santa Clara, CA), transcriptome sequencing was performed on an Illumina HiSeq 3000 platform at Ribobio Co., Ltd. (Guangzhou, Guangdong, China). RNA-seq data sets were processed and analyzed as described previously.^29^ Differentially expressed genes were selected and categorized by Gene Ontology biological process analysis and KEGG pathway enrichment analysis.

### Human ESCC tissue microarray

A human esophageal squamous cell carcinoma tissue microarray was purchased from National Engineering Center Biochip at Shanghai (Shanghai, China), and hematoxylin–eosin staining was performed. The microarray contained 100 tumor tissue and 80 non-tumor tissue, among which 80 tumor tissue and 80 non-tumor tissue were pairs. Original tissue samples were collected from 100 patients who underwent surgery between July 2006 and December 2008, and patient clinicopathological parameters, AJCC 7th staging scores and 6.6–9.0 years of follow-up information were documented. The H-SCORE^30^ was used to semi-quantify stromal HIC-5 staining for each tissue sample, as described previously. The staining intensity was classified into four grades: 1 (negative or trace), 2 (weak), 3 (moderate), and 4 (intense). The total score was the percentage of positive stromal cells (0–100%) multiplied by the intensity of staining, which could be calculated using the formula: 1 × (% of negative cells) + 2 × (% of weak cells) + 3 × (% of moderate cells) + 4 × (% of intense cells). The overall scores of 0– 200, 201–300, and 301–400 were defined as low, medium, and high levels of expression, respectively. Each sample was evaluated in a blinded manner by two senior pathologists, and conflicting cases were reanalyzed by a third pathologist.

### Xenograft tumors derived from ESCC cells mixed with CAFs

Lentivirus vectors for human HIC-5 small hairpin RNA (shRNA) was constructed by Genechem Co., Ltd. (Shanghai, China). The target shRNA sequence is 5′‐GCAGCAGCTTCTTCGAGAA‐3′. Negative control shRNA was provided by Genechem Co., Ltd. (Shanghai, China). Lentivirus‐encoded shRNA against human HIC-5 and control were prepared. CAFs were infected with the prepared lentivirus (MOI = 50). Successful knockdown was confirmed by qRT-PCR and western blot analysis. Fourteen female BALB/c nude mice (6–8 weeks old mice) were randomized in two groups (*N* = 7). KYSE150 cells (4 × 10^6^ cells/mouse) and CAFs-shHIC-5/CAFs-shNC (2 × 10^5^ cells/mouse) were mixed and suspended in PBS and Matrigel at a 1:1 ratio, then injected subcutaneously into right axillary of nude mice. Tumor length (L) and width (W) were measured every other day after 7 days. Tumor volume was calculated using the formula: LW^2^π/6. Tumor weight was measured after mice being killed. The investigator who measured tumor volume and weight was unaware of group allocation. All experiments on animal were given permission by Fudan University School of Basic Medical Sciences.

### Establishment of HIC-5-overexpressing ESCC cell line

Refer to the Supplementary Materials and Methods.

### Statistical analysis

Categorical data for two groups were analyzed using the Chi-square test or Mann–Whitney *U* test; otherwise, a Chi-square test or Kruskal–Wallis H test was used for multiple groups. Continuous data were analyzed using Student’s *t* test for two groups or one-way ANOVA for multiple groups. Time-series experiments were analyzed using two-way ANOVA. Multivariate logistic regression was performed to analyze independent risk factors for lymph node metastasis. All experimental data are represented as the means ± standard error of the mean (SEM). Statistical analysis was performed using SPSS (IBM Corporation, Armonk, NY, USA). *P* < 0.05 was considered statistically significant (two-sided).

## Results

### HIC-5 is highly expressed in CAFs from human ESCC tumor stroma

**Fig. 1:**
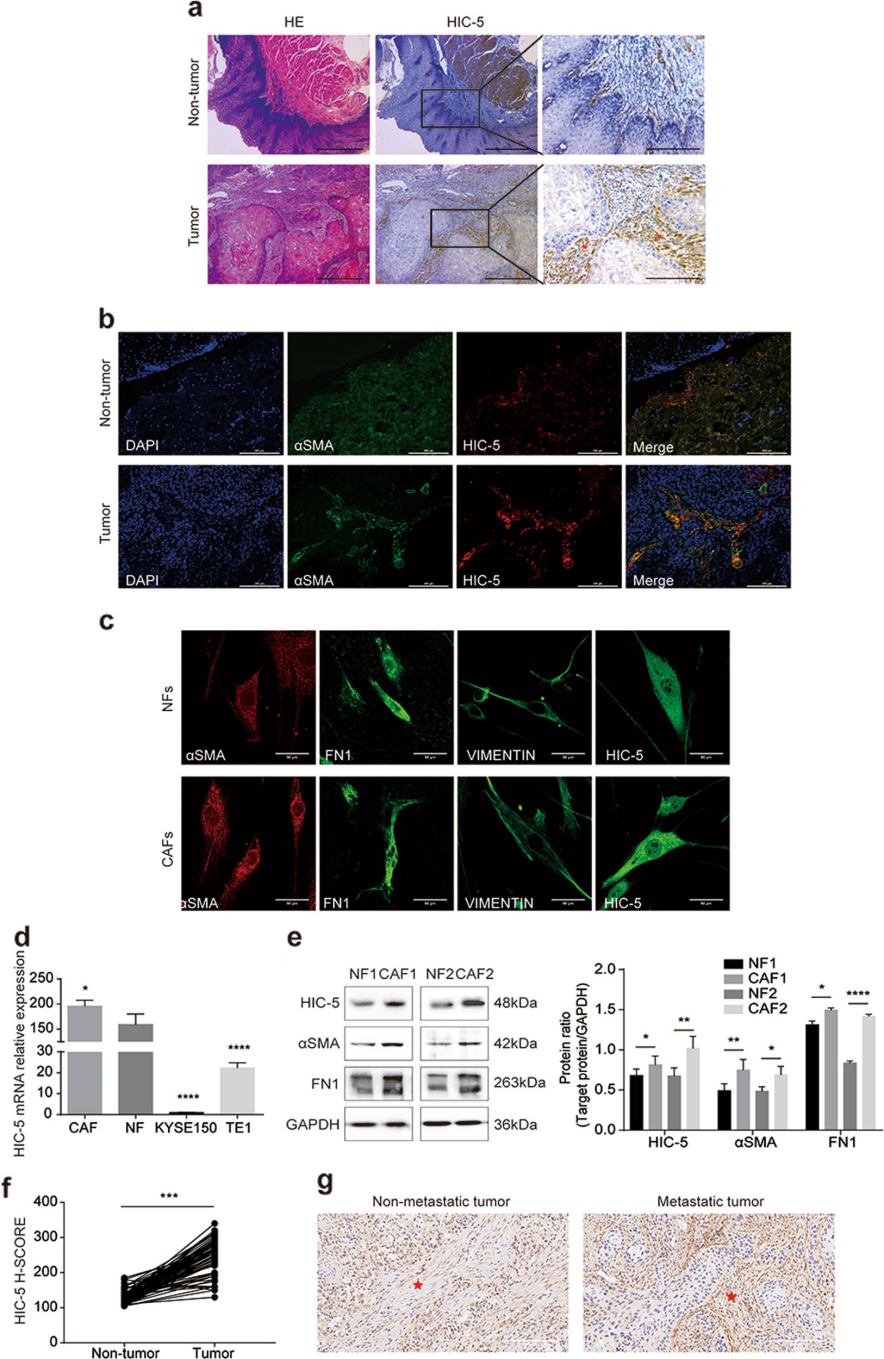
HIC-5 is highly expressed in CAFs from human ESCC tumor stroma. **a** Representative images of hematoxylin–eosin staining and HIC-5 staining in esophageal squamous cell cancer tissue and adjacent non-tumor tissue (scale bar, 500 μm and 200 μm (right panels)). The red arrows represent tumor stroma. **b** Cellular co-localization of αSMA and HIC-5 in ESCC tissue and adjacent non-tumor tissue according to immunofluorescence analysis (scale bar, 200 μm). **c** Identification of isolated CAFs and paired NFs from tumor tissue and adjacent non-tumor tissue according to immunofluorescence staining for αSMA, FN1, and vimentin (scale bar, 50 μm). **d** Relative mRNA expression of HIC-5 in CAFs, NFs, KYSE150, and TE1 cells by qRT-PCR. **e** Western blot analysis of HIC-5, αSMA, and FN1 in NFs and CAFs. **f** Comparison of HIC-5 H-SCORE in 80 pairs of human ESCC tissue and adjacent non-tumor tissue. **g** Representative images of HIC-5 staining in esophageal squamous cell metastatic cancer and nonmetastatic cancer (scale bar, 50 μm). The red asterisks represent tumor stroma. The data represent the mean ± SEM of three independent experiments. *P < 0.05, **P < 0.01, ***P < 0.001, ****P < 0.0001.

We first detected HIC-5 expression in tumor tissue and adjacent non-tumor tissue by immunohistochemistry. As shown in Fig. 1a, HIC-5 was clearly expressed in the stroma of ESCC tissue, while was slightly expressed in the subepithelial stroma of normal esophageal tissue. Co-localization of αSMA and HIC-5 according to immunofluorescence staining revealed that HIC-5 expression was evident in CAFs in the intratumoral stroma (Fig. 1b). To further explore the characteristics of fibroblasts and their interaction with tumor cells, CAFs and paired NFs were isolated from tumor tissue and adjacent non-tumor tissue. Fibroblasts were identified based on positive immunofluorescence staining for αSMA, FN1, and vimentin (Fig. 1c). This result was verified by qRT-PCR (Fig. 1d) and western blot analysis (Fig. 1e), which confirmed that HIC-5 mRNA and protein levels were significantly higher in CAFs than in NFs. Moreover, HIC-5 mRNA levels in KYSE150 and TE1 cells (both are human ESCC cell lines) were very low compared with those in fibroblasts (Fig. 1d). Considering all these findings, we demonstrated that HIC-5 was expressed predominantly in CAFs from cancer stroma.

### Stromal HIC-5 expression is associated with ESCC lymph node metastasis

We performed immunohistochemistry to evaluate the HIC-5 expression levels in esophageal carcinoma stroma and adjacent normal tissue stroma. Consistent with our previous findings, the ESCC tissue microarray revealed that HIC-5 expression levels were higher in tumor stroma than in normal tissue according to the H-SCORE (Fig. 1f). Kaplan–Meier survival analysis of 100 ESCC cases indicated that there were no differences in survival rates among patients with low, medium, or high stromal HIC-5 staining (Supplementary Fig. S1a). However, HIC-5 expression in CAFs seemed to be stronger in metastatic cancer tissue than in nonmetastatic cancer tissue (Fig. 1g). Thus, we speculated that CAF-derived HIC-5 might correlate with ESCC metastasis. Further statistical analysis revealed that increased stromal HIC-5 expression in the tumor stroma was associated with positive lymph node metastasis (*P* = 0.002) and a higher TNM stage (*P* = 0.029), but not with other clinicopathological parameters (Table 1; Supplementary Fig. S1b). Furthermore, univariate analysis showed that lymph node metastasis was related to sex (*P* = 0.009), differentiation (*P* *=* 0.043), and stromal HIC-5 expression (*P* *=* 0.002) (Supplementary Table S2). Male sex (*P* = 0.002), moderate differentiation (*P* *=* 0.015), poor differentiation (*P* *=* 0.013), and high HIC-5 expression in the stroma (*P* = 0.002) were independent risk factors for lymph node metastasis according to multivariate logistic regression analysis (Supplementary Table S3). These analyses suggested that HIC-5 in CAFs contributed to lymph node metastasis in ESCC.

**Table 1:** Relationship between CAF-derived HIC-5 expression and clinicopathological characteristics in 100 ESCC tissues.

Variables	Number	H-score			*P*-value
		Low	Medium	High	
Esophageal cancer	100	20	67	13	
*Gender*					
Male	74	13	54	7	0.802
Female	26	7	13	6	
*Age (year)*					
<60	30	7	20	3	0.471
≥60	70	13	47	10	
*Tumor size (cm)*					
<5	42	6	30	6	0.323
≥5	43	9	30	4	
*Differentiation*					
Well	33	7	22	4	0.937
Moderately	42	7	30	5	
Poorly	25	6	15	4	
*T stage*					
T1 + T2	15	6	8	1	0.06
T3 + T4	82	14	57	11	
*Lymph node metastasis*					
Negative	45	14	29	2	0.002^*^
Positive	54	6	37	11	
*TNM stage*					
I + II	46	13	30	3	0.029^*^
III + IV	50	7	34	9	

*P < 0.05

### HIC-5 in CAFs promotes ESCC cell migration and invasion

**Fig. 2:**
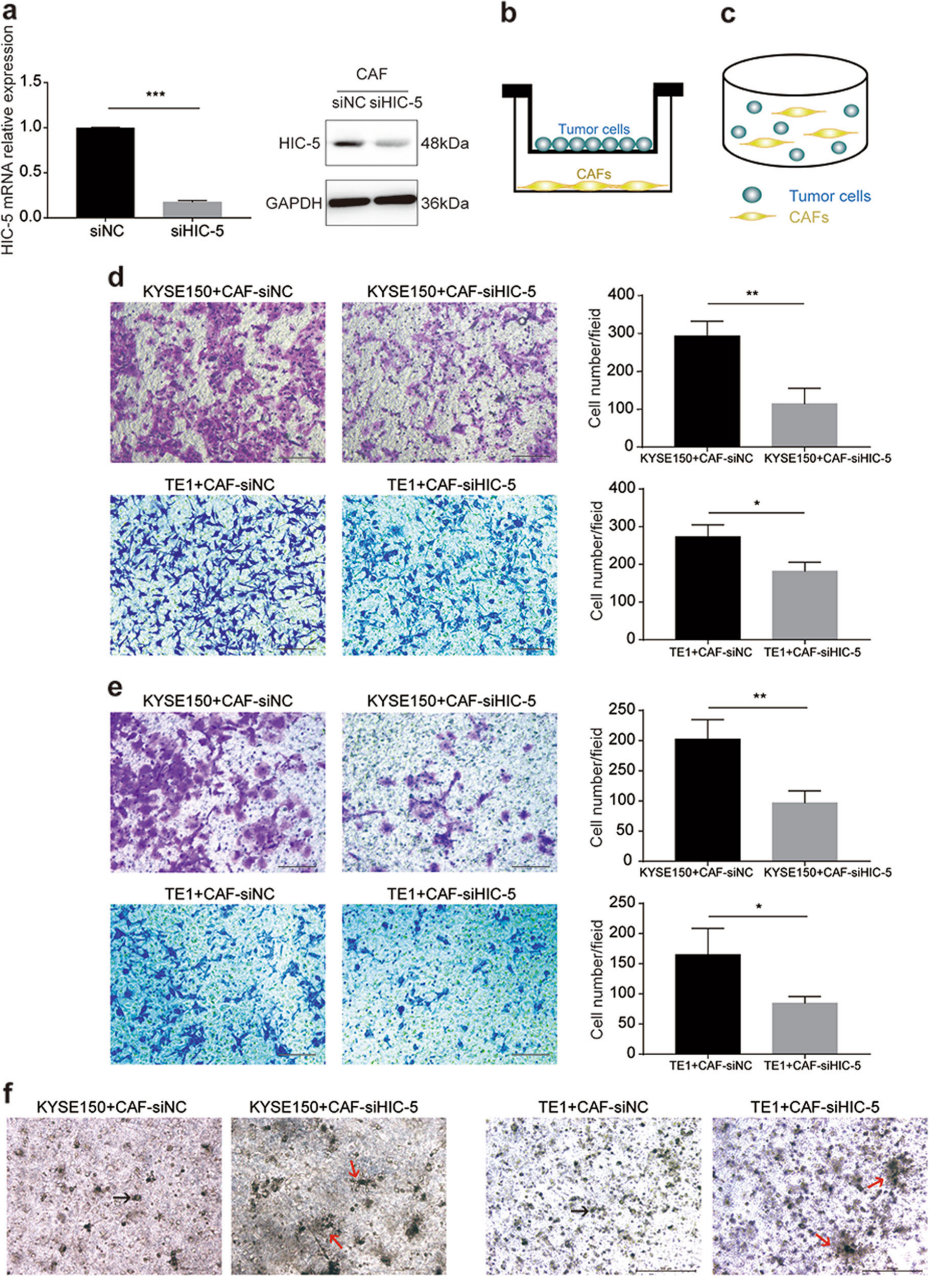
HIC-5 in CAFs promotes ESCC cell migration and invasion. **a** mRNA and protein expression levels of HIC-5 in CAFs detected by qRT-PCR and western blotting, respectively, after transfection with HIC-5-targeting siRNA. **b** Diagram of the indirect co-culture system. **c** Diagram of the direct three-dimensional co-culture system in Matrigel. **d** Representative images of KYSE150 and TE1 cell migration when co-cultured with HIC-5 knockdown CAFs (scale bar, 200 μm). Quantitative analysis of the migrated cells is shown in the right panel. **e** Representative images of KYSE150 and TE1 invasion when co-cultured with HIC-5 knockdown CAFs (scale bar, 200 μm). Quantitative analysis of the invaded cells is shown in the right panel. **f** Representative images of the morphology of KYSE150/TE1 cells co-incubated with CAF-siNC, or with CAF-siHIC-5 (scale bar, 500 μm). The black arrows represent tumor cells colonies. The red arrows represent aggregations of multiple clone formations. The data represent the mean ± SEM of three independent experiments. *P < 0.05, **P < 0.01, ***P < 0.001.

To explore the role of HIC-5 in the regulation of tumor progression, we silenced HIC-5 in CAFs using siRNA. Successful knockdown was confirmed by qRT-PCR and western blot analysis (Fig. 2a). Then, we indirectly co-cultured (separated by semi-membranes) KYSE150/TE1 cells and CAFs in Transwell chambers (with or without Matrigel) for the indicated times (Fig. 2b). Crystal violet staining of the semi-membrane demonstrated decreased tumor cell invasion (Fig. 2d; *P* *<* 0.05) and migration (Fig. 2e; *P* *<* 0.05) after co-culture with HIC-5 knockdown CAFs. We also directly co-incubated KYSE150/TE1 cells and CAFs in Matrigel in vitro (Fig. 2c). When tumor cells and CAFs-siHIC-5 were co-incubated, the aggregation of tumor colonies was more prominent (Fig. 2f). This phenomenon might lead to limited tumor cell migration to distant locations. Taken together, these results suggested that the expression of HIC-5 in CAFs might enhance esophageal cancer cell migration and invasion.

### CAF-derived HIC-5 contributes to tumor progression by regulating cytokines and modifying the ECM

**Fig. 3:**
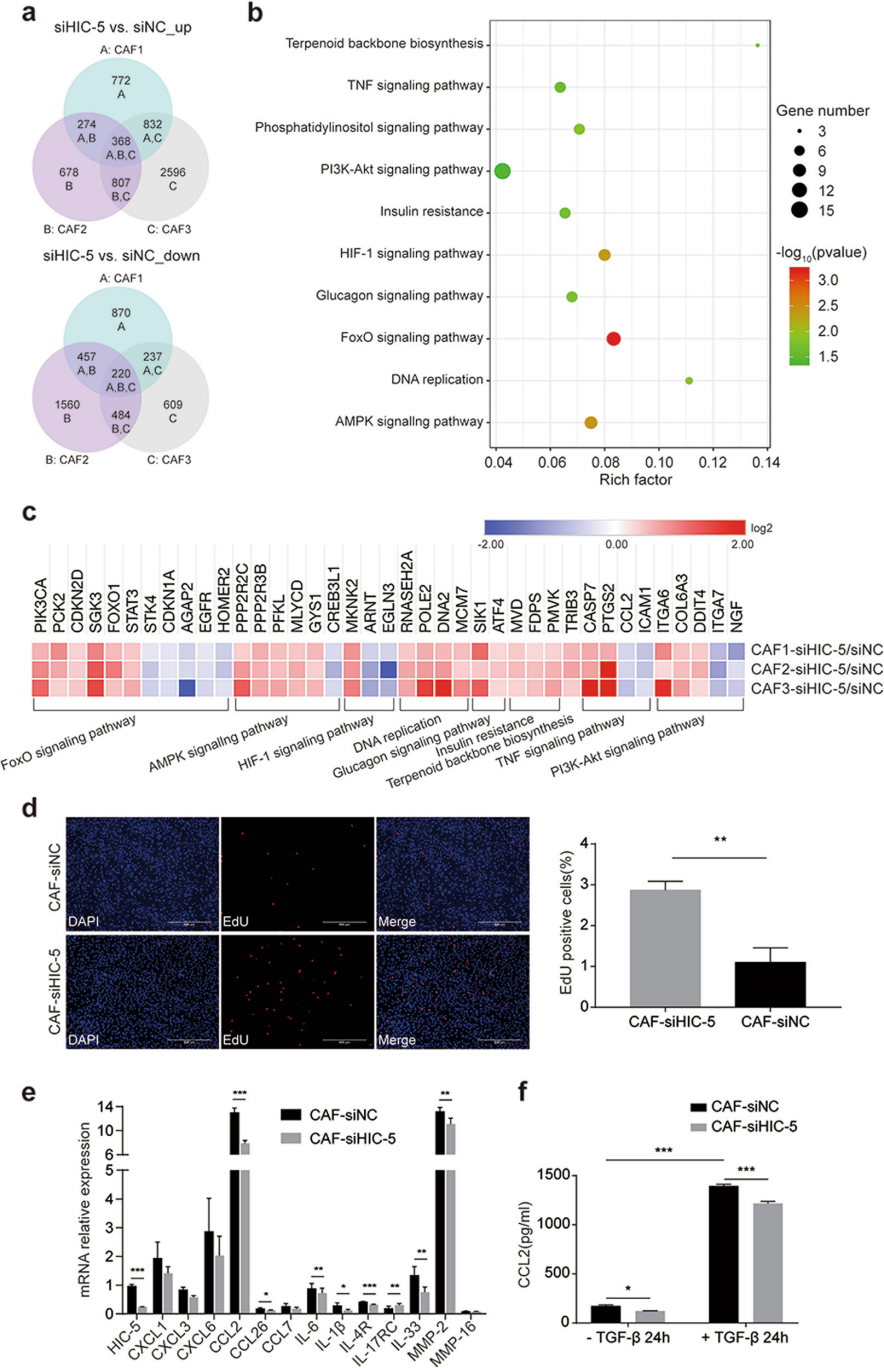
CAF-derived HIC-5 contributes to tumor progression by regulating cytokines and modifying the ECM. **a** Upregulated and downregulated genes in three isolations of HIC-5 knockdown CAFs (fold change ≥ 1.2, *P* < 0.05). **b** Bubble map representing significantly enriched pathways according to KEGG pathway analysis conducted for genes that were upregulated or downregulated. **c** Heatmap representing the log2 fold changes of genes in major pathways different between CAF-siHIC-5 and control CAFs. **d** Representative images of CAF proliferation determined by EdU assay, followed by quantitative analysis of EdU-positive cells (%) (scale bar, 500 μm). **e** Relative mRNA expression levels of selected cytokines/receptors or ECM components that were differentially expressed according to RNA-seq data. **f** Quantitative analysis of supernatant CCL2 levels after TGF-β simulation according to ELISA. The data represent the mean ± SEM of three independent experiments. *P < 0.05, **P < 0.01, ***P < 0.001.

RNA-seq and bioinformatics analyses were performed to analyze differentially expressed genes in HIC-5 knockdown CAFs and control CAFs. Considering the substantial heterogeneity of the expression profile among different patients, the fold change was set to 1.2 to analyze three pairs of CAFs. A total of 368 and 220 genes were identified to be upregulated and downregulated, respectively (Fig. 3a, *P* *<* 0.05). KEGG pathway analysis was conducted on 588 differentially expressed genes. A bubble map shows that these 588 genes were enriched in the FoxO signaling pathway, AMPK signaling pathway, HIF-1 signaling pathway, and DNA replication (Fig. 3b). A heatmap was used to illustrate log2 fold changes in the expression of genes in major pathways between CAF-siHIC-5 and control CAFs (Fig. 3c). Overall, HIC-5 knockdown shifted the CAF gene expression profile to favor proliferation upregulation. We also measured CAF proliferation by EdU assay. CAF-siHIC-5 displayed better proliferation ability than control CAFs (Fig. 3d), which was consistent with the above RNA-seq results.

Fibroblasts play a crucial role in remodeling the ECM and secrete cytokines that contribute to tumor cell migration.^14^ We measured the relative mRNA expression of selected cytokines/receptors or enzymes that were differentially expressed according to the RNA-seq results. As shown in Fig. 3e, qRT-PCR demonstrated that the expression of CCL2, IL-1β, and MMP2 were significantly downregulated in HIC-5 knockdown CAFs. Among these genes, CCL2, also known as MCP-1, is a critical cytokine in recruiting monocytes to the tumor microenvironment, and it is correlated with the progression of several cancers, such as ESCC and colon cancer.^31,32^ We quantified CCL2 levels in the culture medium supernatants of CAFs stimulated with TGF-β, a cytokine secreted by tumor cells and implicated in fibroblast activation. Supernatant CCL2 levels were significantly higher after TGF-β stimulation and, notably, were lower after knocking down HIC-5 expression, independent of TGF-β treatment (Fig. 3f). Together, these findings indicated that HIC-5 in CAFs might contribute to tumor progression by regulating cytokines and modifying the ECM.

### The effects of HIC-5 knockdown in CAFs on xenograft tumors derived from ESCC cells mixed with CAFs

**Fig. 4:**
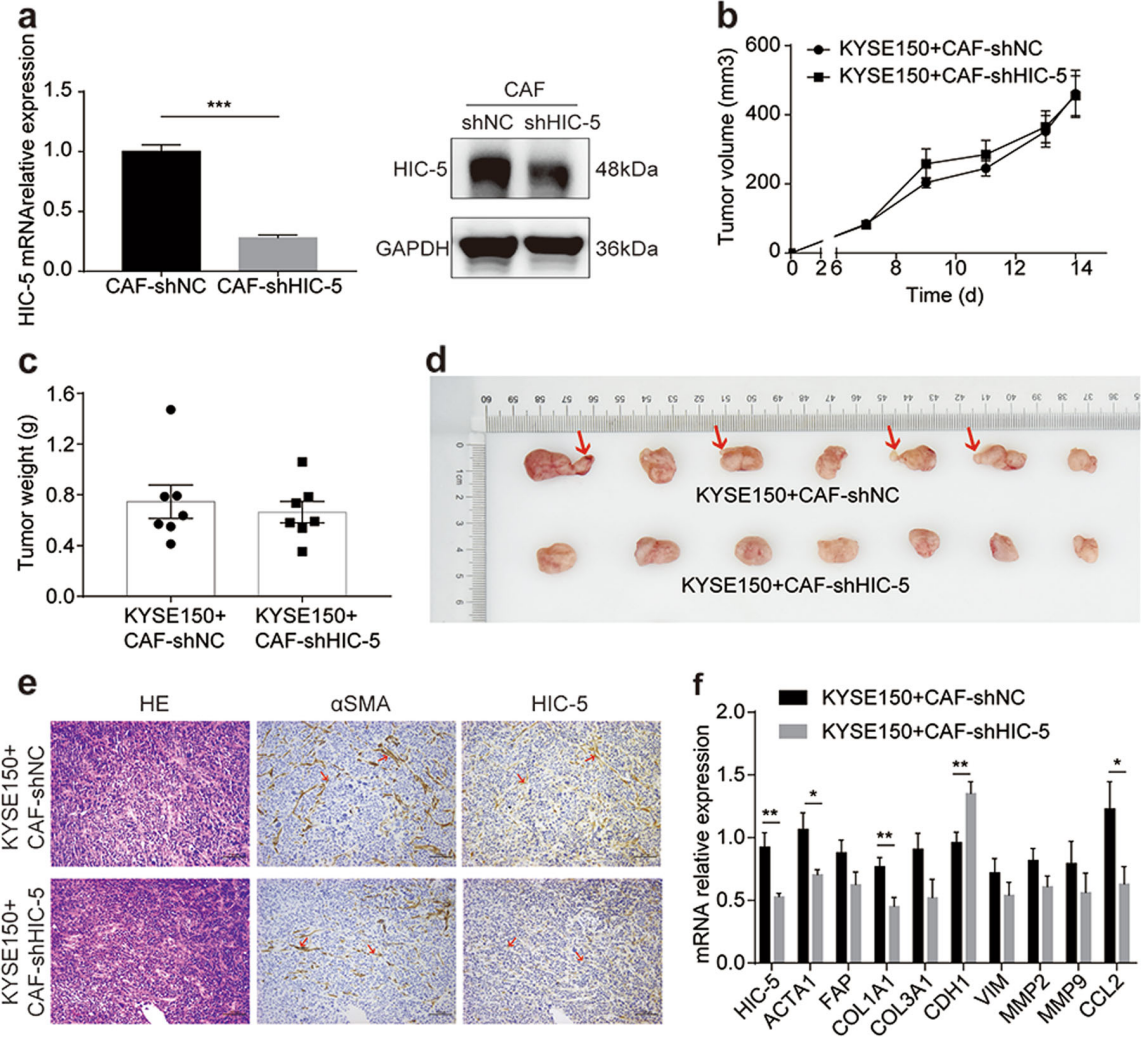
The effects of HIC-5 knockdown in CAFs on xenograft tumors derived from ESCC cells mixed with CAFs. **a** Successful HIC-5 knockdown in CAFs by lentivirus-mediated shRNA, confirmed by qRT-PCR and western blotting. **b** Tumor volume in nude mice derived from KYSE150 cells co-injected with HIC-5 knockdown CAFs or with control CAFs (*N* = 7 per group). **c** Tumor weight derived from KYSE150 cells co-injected with HIC-5 knockdown CAFs or with control CAFs (*N* = 7 per group). **d** Tumors in nude mice from KYSE150 cells co-injected with HIC-5 knockdown CAFs or with control CAFs (*N* = 7 per group). The red arrows represent budding tumor tissue. **e** Representative images of hematoxylin–eosin, HIC-5, and αSMA staining in KYSE150 + CAF-shNC and KYSE150 + CAF-shHIC-5 tumor tissue (scale bar, 100 μm). The red arrows represent CAFs. **f** Relative mRNA expression levels of migration-related genes in KYSE150 + CAF-shNC and KYSE150 + CAF-shHIC-5 tumor tissue (*N* = 7 per group). The data represent the mean ± SEM. *P < 0.05, **P < 0.01, ***P < 0.001.

HIC-5 knockdown CAFs were established utilizing lentivirus-mediated shRNA targeting human HIC-5 (Fig. 4a). KYSE150 were then co-injected with CAFs in nude mice and cultured for 14 days. Both tumor volume and tumor weight showed no statistic difference between the HIC-5 knockdown CAFs group and the control group (Fig. 4b, c). The tumors from the CAF-shHIC-5 group had grossly less irregular borders and presented more regular morphology, while the budding tumor tissue were observed in the control group (Fig. 4d), which might be a sign of its invasiveness. Histological study revealed that HIC-5 expression was lower in tumors from CAF-shHIC-5 group than that in tumors from CAF-shNC group (Fig. 4e). Further qRT-PCR revealed that COL1A1 was decreased and CDH1, an invasive suppressor gene, was elevated in CAF-shHIC-5 group comparing with the control group (Fig. 4f). Besides, in consistence with prior observation, CCL2 expression in tumors was also downregulated when knocking down HIC-5 in CAFs.

### HIC-5 has distinct biological functions in CAFs vs. NFs, especially in cell movement and the Rho GTPase signaling pathway

**Fig. 5:**
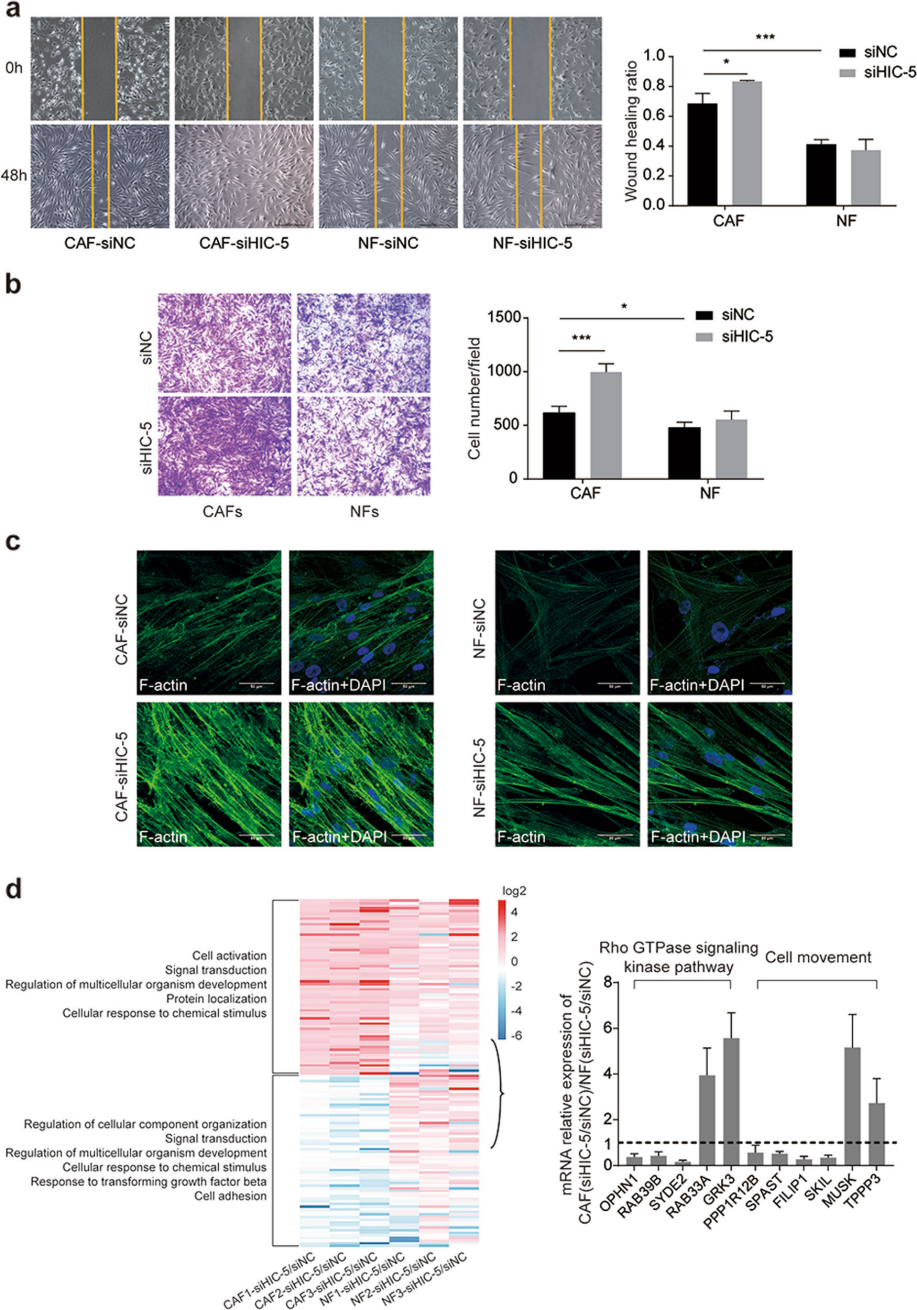
HIC-5 has distinct biological functions in CAFs vs. NFs, especially in cell movement and the Rho GTPase signaling pathway. **a** Representative image of CAFs/NFs (with or without HIC-5 knockdown) in wound-healing assays at 48 h (scale bar, 500 μm). Quantitative analysis of the wound-healing ratio is shown in the right panel. **b** Representative images of CAF/NF (with or without HIC-5 knockdown) migration at 48 h (scale bar, 500 μm). Quantitative analysis of cell migration is shown in the right panel. **c** Immunofluorescence staining for F-actin by phalloidin in CAFs/NFs (with or without HIC-5 knockdown) (scale bar, 50 μm). **d** Heatmap representing the comparison of gene expression changes upon HIC-5 knockdown in CAFs vs. NFs. Right panel: the relative mRNA expression above the dashed line indicates more apparent upregulation in CAFs than in NFs when HIC-5 is knocked down; the relative mRNA expression below the dashed line indicates more apparent downregulation in HIC-5 knockdown CAFs than in NFs. The data represent the mean ± SEM of three independent experiments. *P < 0.05, ***P < 0.001.

Tumors are considered “wounds that do not heal”.^33^ Fibroblasts react when cancer cells accumulate in tissue, similar to the chronic wound-healing process, which requires fibroblast recruitment and migration. Consequently, we next performed a cell movement ability assay with CAFs and NFs and compared the results. CAFs had a higher migration rate than NFs. HIC-5 knockdown increased the cell migration rate in CAFs but did not have the same impact on NFs, which was demonstrated through both wound-healing assays (Fig. 5a) and Transwell assays (Fig. 5b). HIC-5 knockdown in CAFs substantially enhanced the expression of F-actin, as would be expected in the case of increased mobility (Fig. 5c). To investigate why HIC-5 has distinct functions in CAFs vs. NFs, an additional RNA-seq analysis was conducted for NF-siHIC-5. For a fold change >1.5 (*P* < 0.05), 67 upregulated genes and 66 downregulated genes were identified when comparing CAF-siHIC-5 to control CAFs. The degree of alteration of these 133 genes was then juxtaposed with their counterparts in NF-siHIC-5 vs. control NFs, as shown in the heatmap (Fig. 5d). CAFs and NFs demonstrated a distinct pattern of expression alterations upon HIC-5 knockdown. Eleven genes with the most significant differences between the two groups were further analyzed by qRT-PCR to confirm their relative mRNA expression. A Gene Ontology review revealed that these genes were enriched in the Rho GTPase signaling pathway and cell movement (Fig. 5d).

### HIC-5 has distinct effects on TGF-β signaling and the Rho GTPase signaling kinase pathway in CAFs and NFs

**Fig. 6:**
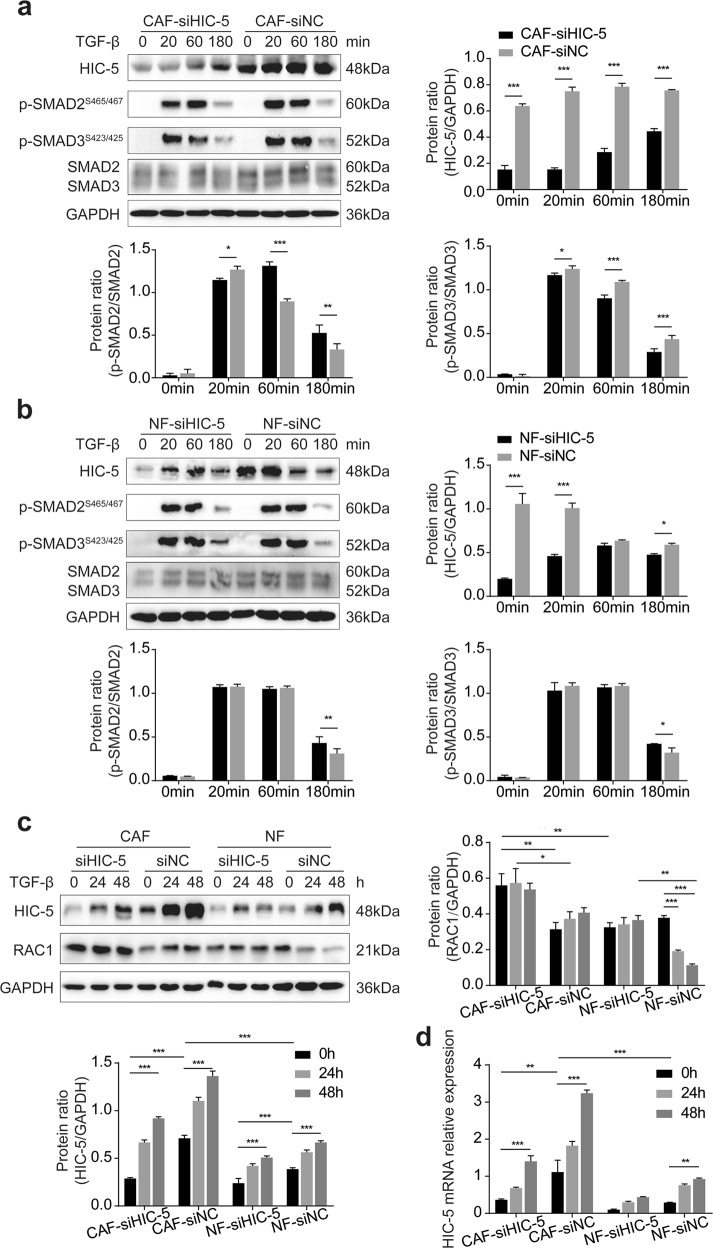
HIC-5 has distinct effects on TGF-β signaling and the Rho GTPase signaling pathway in CAFs and NFs. **a** Western blot analysis for p-SMAD2, p-SMAD3, SMAD2, SMAD3, and HIC-5 expression in CAF-siHIC-5 vs. CAF-siNC in response to TGF-β treatment for 0, 20, 60, and 180 min. **b** Western blot analysis for p-SMAD2, p-SMAD3, SMAD2, SMAD3, and HIC-5 expression in NF-siHIC-5 vs. NF-siNC in response to TGF-β treatment for 0, 20, 60, and 180 min. **c** Western blot analysis for RAC1 and HIC-5 expression in response to TGF-β treatment for 0, 24, and 48 h in CAFs vs. NFs with or without HIC-5 knockdown. **d** Relative mRNA expression of HIC-5 in response to TGF-β treatment for 0, 24, and 48 h in CAFs vs. NFs with or without HIC-5 knockdown. The data represent the mean ± SEM of three independent experiments. *P < 0.05, **P < 0.01, ***P < 0.001.

Considering the close relationship between HIC-5 and TGF-β, we further explored how HIC-5 affects the activation of TGF-β signaling and the Rho GTPase signaling kinase pathway upon TGF-β stimulation. In both CAFs and NFs, the phosphorylation of both SMAD2 and SMAD3 was notably augmented after TGF-β exposure (Fig. 6a, b). However, in CAFs, there was a difference in the pattern of increased phosphorylation for SMAD2 and SMAD3: the increase in SMAD2 phosphorylation was more significant for CAFs-siHIC-5 than for CAF-siNC; on the contrary, HIC-5 knockdown prevented the increase in SMAD3 phosphorylation (Fig. 6a). This effect was not observed in NFs, as shown in Fig. 6b, where the change in SMAD2/3 phosphorylation was the same, regardless of whether HIC-5 was knocked down.

We then evaluated HIC-5 and RAC1 expression after TGF-β exposure for 24 and 48 h, and the results showed that HIC-5 levels increased in both CAFs and NFs, regardless of HIC-5 knockdown (Fig. 6c, d). RAC1 expression was dramatically higher in HIC-5 knockdown CAFs than in control CAFs, but was not different between the two NF groups (Fig. 5c). All these findings implied that HIC-5 had distinct effects on TGF-β signaling and the Rho GTPase signaling pathway in CAFs and NFs.

### The role of HIC-5 in tumor parenchymal cells

HIC-5 is also expressed in tumor cells, as shown in the tissue microarray (Supplementary Fig. S2a). Sixteen of 100 ESCC cases were HIC-5 positive; however, positive status did not correlate with the aforementioned clinicopathological parameters. HIC-5 was then successfully overexpressed in KYSE150 and TE1 cells by lentivirus transfection (Supplementary Fig. S2b). The effect of HIC-5 on key determinants of cancer progression, including cell proliferation, migration, and invasion, was assessed. As shown in Supplementary Fig. S2c, cell proliferation, as determined by EdU assay, was evidently increased when HIC-5 was overexpressed in KYSE150 cells. Similarly, according to the CCK-8 assay results, HIC-5 overexpression led to a higher cell growth rate in both KYSE150 and TE1 cells (Supplementary Fig. S2d). Interestingly, cell migration (Supplementary Fig. S2e) and invasion (Supplementary Fig. S2f) both decreased in HIC-5-overexpressing KYSE150 and TE1 cells. Similarly, we used RNA-seq analysis to investigate the altered expression of downstream genes in HIC-5-overexpressing KYSE150 cells. Overall, 906 genes were obviously changed, including 584 upregulated genes and 322 downregulated genes (Supplementary Fig. S2g; over a twofold change, *P* < 0.05). The biological processes of these genes were enriched in “regulation of cell proliferation”, “cytokine production”, “cell death”, and “regulation of cell motility” (Supplementary Fig. S2h). In brief, our study implied that HIC-5 played distinct yet overlapping roles in esophageal cancer cells and fibroblasts.

## Discussion

Except for dysphagia, warning signs and age have limited predicted value for detecting ESCC.^34^ Patients are often diagnosed at a late stage of cancer when dysphagia occurs, which results in poor outcomes. CAFs play an important role in cancer invasion and metastasis, but the underlying mechanisms have not been fully elucidated. Herein, we demonstrated that HIC-5 was highly expressed in tumor stroma of human ESCC, and specifically, CAF-derived HIC-5 contributed to esophageal cancer cell migration and invasion.

No publication has yet reported the function of HIC-5 in ESCC. We observed that HIC-5 in CAFs enhances the migration and invasion of cancer cells. The correlation between lymph node metastasis and high levels of HIC-5 expression was also confirmed by tissue microarray. Increased HIC-5 expression in the tumor stroma was associated with an independent risk factor for lymph node metastasis. These results were similar to those of a previous study in which HIC-5 overexpression was correlated with intra- and extra-hepatic metastasis in human hepatocellular carcinoma.^23^ Although we did not observe tumor metastasis in vivo directly, the mRNA expression data and xenograft tumor morphology showed that CAF-derived HIC-5 led to a tendency to metastasize. Goreczny et al. reported that increased HIC-5 expression in breast tumors was not related to reduced distant metastasis-free survival when all tumor types were considered, but was correlated with poor outcomes for HER2 + breast tumors.^26^ In this study, we did not observe a correlation between overall survival and the stromal HIC-5 expression level. A larger sample size and pertinent genotypical information are required to further characterize the impact of CAF-derived HIC-5 on ESCC survival, especially among varied genotypic subtypes.

TGF-β is a well-recognized factor that contributes to the phenotypic switch of NFs to CAFs, and is a key mediator of fibroblast proliferation, recruitment, and invasion. As key mediators of the TGF-β superfamily signaling pathway,^35,36^ Phosphorylated SMAD2 and SMAD3 translocate to the nucleus and regulate target genes with other nuclear cofactors. SMAD2 increases TGF-β1-induced growth inhibition, while SMAD3 inhibits growth inhibition and migratory responses.^37^ Moreover, SMAD2 depletion enhances SMAD3 activity and vice versa, indicating a dynamic balance between these two mediators to regulate cell activities.^38,39^ We found that SMAD2 phosphorylation was increased and that SMAD3 phosphorylation was decreased when HIC-5 was knocked down in CAFs, whereas SMAD2/3 phosphorylation was not significantly different in NFs. These results suggest that HIC-5 impedes the movement of CAFs through TGF-β/SMAD signaling.

RAC1, an important member of the Rho GTPase family, is involved in many cellular processes, including the cell cycle, cytoskeleton remodeling, cell motility, and cell adhesion. Overexpression of RAC1 in cancer cells induces lamellipodia formation and promotes abnormal cell movement, which may lead to epithelial–mesenchymal transition (EMT).^40–42^ In MCF10A cells, HIC-5 acts as a crucial mediator of TGF-β-stimulated invadopodia formation and regulates matrix degradation and invasion during EMT as upstream effector of the RAC1-p38 MAPK pathway.^43^ A similar role of HIC-5 was also observed in MDA-MB-231 breast cancer cells, where HIC-5 downregulation caused a decrease in RAC1 and possibly triggered cell invasion.^21^ However, HIC-5 reduces EGF-dependent lamellipodia formation by inhibiting RAC activation in COS-7 cells.^44^ These distinct functions indicate that HIC-5 may play bidirectional roles in regulating RAC1 activity in epithelial cells and stromal cells. We identified that RAC1 was dramatically upregulated in HIC-5 knockdown CAFs compared with that in control CAFs, but displayed little if any differences between the two NF groups. This finding is consistent with the alteration of TGF-β/SMAD signaling, which implies that HIC-5 can inhibit the motility of CAFs and has dissimilar functions in CAFs and NFs.

Several metabolic pathways were altered according to the RNA-seq analysis comparing HIC-5 knockdown CAFs and control CAFs. Some epithelial cancer cells can induce the Warburg effect in neighboring stromal fibroblasts, which is termed the “Reverse Warburg Effect”. CAFs differentiate into myofibroblasts and produce lactate and pyruvate through aerobic glycolysis. Energy-rich metabolites can be taken up by cancer cells and used in the mitochondrial TCA cycle. Efficient ATP production creates the proper environment for cancer cell proliferation.^45^ Our RNA-seq data showed that silencing HIC-5 in CAFs altered several metabolic pathways, including the FoxO signaling pathway and the AMPK signaling pathway, which indicates that HIC-5 may participate in crosstalk between CAFs and cancer cells by influencing the production of metabolites. On the other hand, CAF-derived HIC-5 enhances the secretion of cytokines such as CCL2. The presence of pro-inflammatory cytokines stimulates glycolysis,^46–48^ which may lead to energy-rich metabolites locally. In essence, through regulating metabolic signaling pathways and promoting the inflammatory state, CAF-derived HIC-5 may provide metabolites for cancer cells by upregulating CAF glycolysis and thereby accelerate cancer progression.

To the best of our knowledge, there has been no study comparing the role of HIC-5 in CAFs and NFs. RNA-seq analysis and cell experiments both indicated distinct functions of HIC-5, especially related to cell movement. HIC-5 attenuates CAF movement, which might limit its ability to surround cancer cells to restrict cancer migration. NFs did not demonstrate significant changes in cell movement abilities upon HIC-5 interference, likely due to their quiescent status in the absence of cancer cell stimulation. Intriguingly, TGF-β induced notable HIC-5 accumulation in the nuclei of NFs, but not in CAFs (Supplementary Fig. S3). As a shuttle between focal adhesions and the nucleus, HIC-5 relays signaling from the cytoplasm to the nucleus. Nuclear HIC-5 in NFs was reported to induce LOX expression and was responsible for tumorigenesis in colorectal cancer.^28^ Nuclear HIC-5 also works as a stromal-specific coactivator of androgen receptor (AR) to regulate AR target genes in prostate tumors, which then creates a microenvironment conducive to tumor growth and progression.^49^ The nuclear accumulation of HIC-5 in NFs but not in CAFs may indicate that nuclear HIC-5 transduces signals and lays the foundation for early tumorigenesis, while cytoplasmic HIC-5 mainly contributes to cancer progression.

We also explored the role of HIC-5 in parenchymal cells, where its expression is significantly lower than that in the stroma. HIC-5 appears to enhance cancer cell proliferation, but decrease migration and invasion in KYSE150 cells. These results are not consistent with those of previous studies, which reported that HIC-5 enhanced EMT in breast cancer and prostate cancer cells, as well as promoted metastasis in breast cancer and hepatocellular carcinoma.^21,23,50^ In our tissue microarray, no correlation was shown between HIC-5 expression in ESCC cells and cancer progression, which suggests that HIC-5 contributes to ESCC progression mainly through the medium of CAFs. Nevertheless, these discrepancies may also represent the heterogeneity of cancer behavior. Further experiments are required to clarify these observations.

In summary, we identified that stromal HIC-5 was a predictive factor for lymph node metastasis in human ESCC and that CAF-derived HIC-5 regulated ESCC cell migration and invasion by regulating cytokines and modifying the ECM. These findings provide a foundation for further understanding the mechanism by which HIC-5 regulates cytokines and implicate CAF-derived HIC-5 as a potential therapeutic target.

## Supplementary information

supplementary (method, Table S1–3)

Supplementary Figure Legends

Figure S1

Figure S2

Figure S3

## Data Availability

Raw data of RNA sequencing that support the findings of this study have been deposited in SRA database of NCBI with the accession number PRJNA575222.
