# Supplementary material for: HIC-5 in cancer-associated fibroblasts contributes to esophageal squamous cell carcinoma progression
Source: Cell Death Dis. 2019 Nov 18;10(12):873. doi: 10.1038/s41419-019-2114-z (PMC6861248; doi:10.1038/s41419-019-2114-z)
Supplement: Supplementary file 1 — supplementary (method, Table S1–3) [file 41419_2019_2114_MOESM1_ESM.docx]

**Materials and methods**

**Establishment of HIC-5-overexpressing ESCC cell line**

Lentiviral vectors containing human *HIC-5* (Genechem Co., Ltd., Shanghai, China) were co-transfected with packaging plasmids pGag-Pol, pRev and pVSVG into HEK293T cells. Supernatant containing lentiviruses was harvested, filtered and concentrated at 24, 48 and 72 h after transfection. KYSE150 and TE1 cells were infected with lentiviruses for 48 h, followed by treatment with puromycin for selection. An empty vector was transfected into KYSE150 and TE1 cells as a control.

**Tables**

**Table S1** Primer sets used for qRT-PCR

| **Gena name** | **Primer** | **Sequence (5’-3’)** |
| --- | --- | --- |
| HIC-5 | Forward | GGGCAAGTGGTGACGGCT |
|  | Reverse | TGTGTCGGATGGGCTGGTT |
| 36B4 | Forward | AGCCACGCTGCTGAACAT |
|  | Reverse | CAACATTGCGGACACCCT |
| CXCL1 | Forward | AAGAACATCCAAAGTGTGAACG |
|  | Reverse | CACTGTTCAGCATCTTTTCGAT |
| CXCL3 | Forward | GCCCAAACCGAAGTCATAG |
|  | Reverse | GGTGCTCCCCTTGTTCAGTA |
| CXCL6 | Forward | TGAGAGTAAACCCCAAAACGAT |
|  | Reverse | CAAACTTGCTTCCCGTTCTTC |
| CCL2 | Forward | GCTCATAGCAGCCACCTT |
|  | Reverse | GGAATCCTGAACCCACTT |
| CCL26 | Forward | GGAGTGACATATCCAAGACCTG |
|  | Reverse | CTTGGATGGGTACAGACTTTCT |
| CCL7 | Forward | AACTACCTGCTGCTACAGATTT |
|  | Reverse | CTTGTCCAGTTTGGTCTTGAAG |
| IL-6 | Forward | CACTGGTCTTTTGGAGTTTGAG |
|  | Reverse | GGACTTTTGTACTCATCTGCAC |
| IL-1β | Forward | GCCAGTGAAATGATGGCTTATT |
|  | Reverse | AGGAGCACTTCATCTGTTTAGG |
| IL-4R | Forward | TACTTGCGAGTGGAAGATGAAT |
|  | Reverse | TATAGTTATCCGCACTGACCAC |
| IL-17RC | Forward | GAATCTGGTCCTATACTCAGCC |
|  | Reverse | GTGGTTCAAGGTAATGATCTGC |
| IL-33 | Forward | GCTTTGCCTTTGGTATATCAGG |
|  | Reverse | CTGATTCATTTGAGGGGTGTTG |
| MMP-2 | Forward | ATTGTATTTGATGGCATCGCTC |
|  | Reverse | ATTCATTCCCTGCAAAGAACAC |
| MMP-16 | Forward | GTTTGGTTACAAAAGTACGGCT |
|  | Reverse | GTTTCTGTCCACTTTTCCTGTC |
| OPHN1 | Forward | GAAAGAGACCAAAACACTGACC |
|  | Reverse | CCTTCTCACACAGTACTTCAGT |
| RAB39B | Forward | GAACTCAGTAGGTGGTCTTCTC |
|  | Reverse | AACGTGTACTTTGGTCTCTTCT |
| SYDE2 | Forward | CGTCTTTTGTGCAATTCAGGTA |
|  | Reverse | TGGGTTCCCAACTGAATACTAC |
| RAB33A | Forward | GAGAAGATCAAGGTTCAGGTGT |
|  | Reverse | CATGTACGTTGCGGTAGTAATG |
| GRK3 | Forward | GAGGGAAACAATTTGTCTTGCA |
|  | Reverse | CCTTGAAGGTTTCGTTCAACTC |
| PPP1R12B | Forward | CAAGAGGTGCTGATATCAACAC |
|  | Reverse | TTGTCTTGCTGGTTTACATTGG |
| SPAST | Forward | CTATTTCTCCTACCCGCTGTTT |
|  | Reverse | CTTATACCATTCCACAGCTTGC |
| FILIP1 | Forward | CAAGAGCATGAAGAGATGAACG |
|  | Reverse | GTCTCTTCTAGCTCCTCGATTC |
| SKIL | Forward | GATAAACAAGGTGCCAACAGTT |
|  | Reverse | AGAAGTACAGGTTCGCTTAACA |
| MUSK | Forward | ACACTACAGAGTCTGATGTGTG |
|  | Reverse | CGTTCCAGAATTCGGTGAATAC |
| TPPP3 | Forward | CCAAGAGATTCAAGGGGAAGAG |
|  | Reverse | CCCCTGTTTTTGCTTTAGTGAC |

**Table S2.** Univariate analysis of risk factors of lymph node metastasis

| **Variables** | **Number** | **Lymph node metastasis** | | ***P* value** |
| --- | --- | --- | --- | --- |
|  |  | Negative | Positive |  |
| **Esophageal Cancer** | 99 | 45 | 54 |  |
| **Gender** |  |  |  |  |
| male | 74 | 28 | 46 | 0.009^*^ |
| female | 25 | 17 | 8 |  |
| **Age (year)** |  |  |  |  |
| <60 | 29 | 10 | 19 | 0.158 |
| ≥60 | 70 | 35 | 35 |  |
| **Tumor size (cm)** |  |  |  |  |
| <5 | 41 | 20 | 21 | 0.394 |
| ≥5 | 43 | 17 | 26 |  |
| **Differentiation** |  |  |  |  |
| Well | 32 | 20 | 12 | 0.043^*^ |
| Moderately | 43 | 16 | 27 |  |
| Poorly | 24 | 9 | 15 |  |
| **T Stage** |  |  |  |  |
| T1+T2 | 15 | 9 | 6 | 0.197 |
| T3+T4 | 81 | 34 | 47 |  |
| **HIC-5 H-SCORE** |  |  |  |  |
| low | 20 | 14 | 6 | 0.002^*^ |
| medium | 66 | 29 | 37 |  |
| high | 13 | 2 | 11 |  |

**P* < 0.05

**Table S3.** Multivariate logistic regression analysis of risk factors of lymph node metastasis

| **Variables** | **OR** | **95.0% *CI* for OR** | | ***P* value** |
| --- | --- | --- | --- | --- |
|  |  | Lower | Upper |  |
| **Gender** |  |  |  |  |
| male | 1.000 |  |  |  |
| female | 0.123 | 0.033 | 0.457 | 0.002^*^ |
| **Differentiation** |  |  |  |  |
| Well | 1.000 |  |  |  |
| Moderately | 3.858 | 1.299 | 11.457 | 0.015^*^ |
| Poorly | 5.467 | 1.424 | 20.990 | 0.013^*^ |
| **HIC-5 H-SCORE** |  |  |  |  |
| low | 1.000 |  |  |  |
| medium | 2.871 | 0.863 | 9.550 | 0.086 |
| high | 27.041 | 3.411 | 214.362 | 0.002^*^ |

**P* < 0.05
