# Supplementary material for: HIC-5 in cancer-associated fibroblasts contributes to esophageal squamous cell carcinoma progression
Source: Cell Death Dis. 2019 Nov 18;10(12):873. doi: 10.1038/s41419-019-2114-z (PMC6861248; doi:10.1038/s41419-019-2114-z)
Supplement: Supplementary file 2 — Supplementary Figure Legends [file 41419_2019_2114_MOESM2_ESM.docx]

**Legends**

**Figure S1.** Stromal HIC-5 expression is associated with ESCC lymph node metastasis. **a** Kaplan-Meier survival curves in human ESCC according to HIC-5 staining (N = 100 patients). **b** Proportions of stromal HIC-5 staining levels according to lymph node metastasis (left panel, N = 99 patients) and TNM stage (right panel, N = 96 patients).

**Figure S2.** The role of HIC-5 in tumor parenchymal cells. **a** Representative image of HIC-5 staining in ESCC tissue. The red arrows represent tumor cells (scale bar, 50μm). The black asterisk represents tumor stroma. **b** Successful HIC-5 overexpression in KYSE150 and TE1 confirmed by qRT-PCR and western blotting. **c** Representative images of KYSE150 proliferation determined by EdU assay, followed by quantitative analysis of EdU positive cells (%) (scale bar, 500μm). **d** The effect of HIC-5 overexpression on KYSE150 and TE1 cell growth was measured by CCK-8 assay. **e** Representative images of KYSE150 and TE1 migration, followed by counts of migrated cells (scale bar, 500μm). **f** Representative images of KYSE150 and TE1 invasion, followed with counts of invasive cells (scale bar, 100μm). **g** Upregulated and downregulated genes in HIC-5 overexpressing KYSE150. **h** Enriched biological process category by Gene Ontology analysis concerning differentially expressed genes between KYSE150-HIC-5 and KYSE150-con. The data represent the mean ± SEM of three independent experiments. **P* <0.05, ***P* <0.01, ****P* <0.001

**Figure S3.** TGF-β induced notable HIC-5 accumulation in the nuclei of NFs. Immunofluorescence staining for HIC-5 in CAFs/NFs (with or without TGF-β stimulation) (scale bar, 50μm). The white arrows represent HIC-5 accumulation in the nuclei.

**Table S1** Primer sets used for qRT-PCR

**Table S2.** Univariate analysis of risk factors of lymph node metastasis

**Table S3.** Multivariate logistic regression analysis of risk factors of lymph node metastasis
