# Supplementary figures and images for: HIC-5 in cancer-associated fibroblasts contributes to esophageal squamous cell carcinoma progression
Source: Cell Death Dis. 2019 Nov 18;10(12):873. doi: 10.1038/s41419-019-2114-z (PMC6861248; doi:10.1038/s41419-019-2114-z)

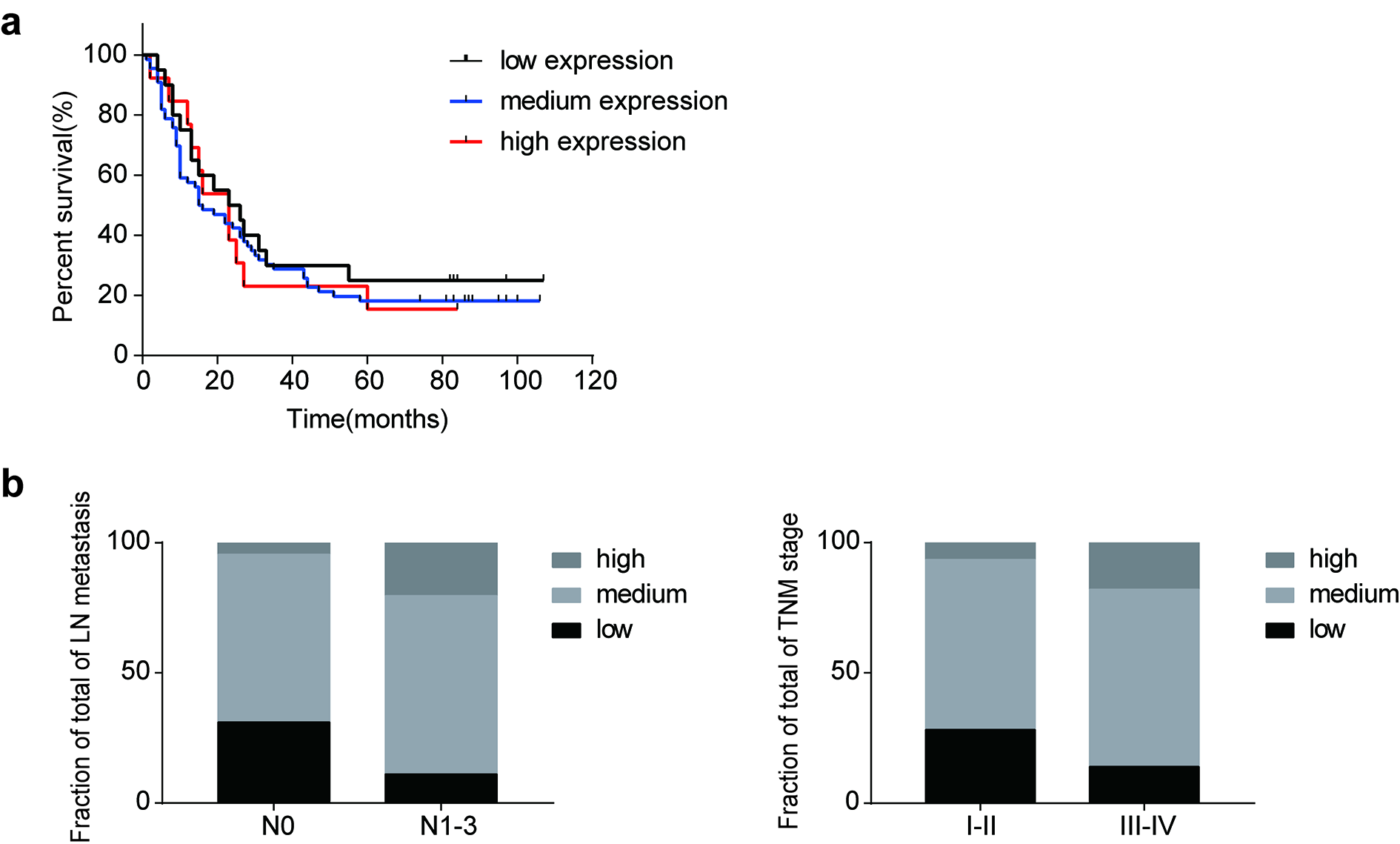

Supplement: Supplementary file 3 — Figure S1 [file 41419_2019_2114_MOESM3_ESM.tif]

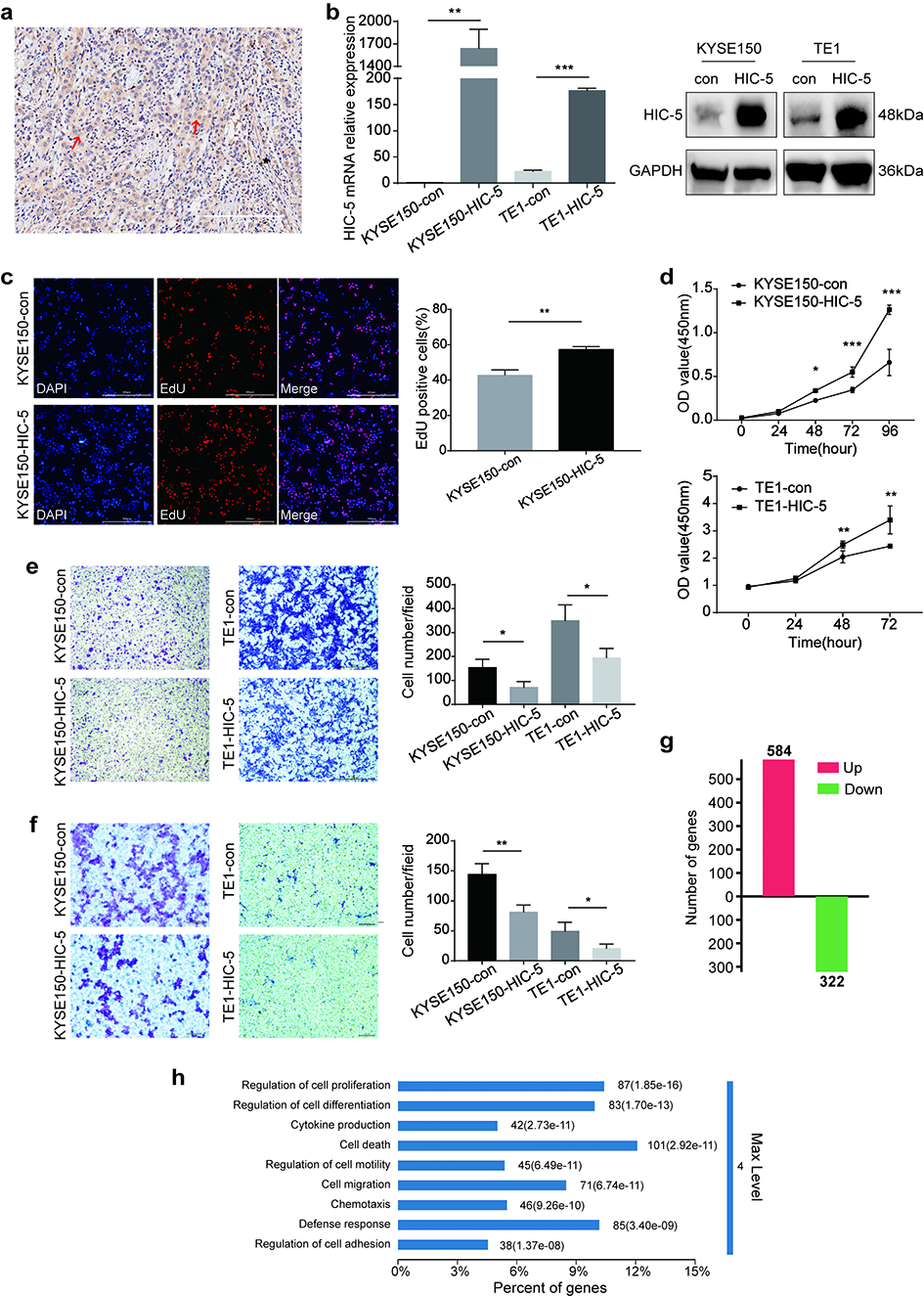

Supplement: Supplementary file 4 — Figure S2 [file 41419_2019_2114_MOESM4_ESM.tif]

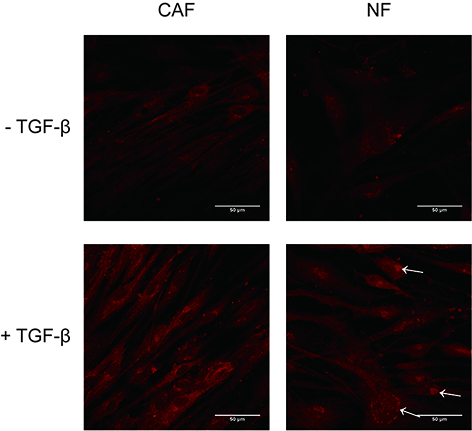

Supplement: Supplementary file 5 — Figure S3 [file 41419_2019_2114_MOESM5_ESM.tif]
